# Medical Opinion and Movement

**Published:** 1909-09-18

**Authors:** 


### Medical Opinion and Movement.

Lesions of the Optic Nerve as an early sign of Disseminated Sclerosis forms the subject of an interesting contribution to the *Archives of Internal Medicine* by Dr. A. Gordon. In the last seven years he has had 56 cases of this disease under his observation, and in 23 of these, or 41 per cent., lesions of the optic nerve were present. This would appear to show a much more frequent occurrence of optic nerve lesions than is usually experienced in this disease, but the chief point of interest lies in the fact that according to the observations of Dr. Gordon this lesion was in a certain number, and probably in a large number of the cases, the first and for some time the only sign of the disease. In 18 of the 23 cases all the typical symptoms of the disease were already established, together with optic atrophy or retro-bulbar neuritis, but in eight of them, by a close questioning, the author was led to the conclusion that some sort of vision trouble formed one of the earliest signs of the disease. In the remaining five eases the author was able to observe the onset of the disease with ocular affections, and in three cases has watched the subsequent development of the malady, although the classic symptoms did not appear for some time afterwards—in one case four years, in another two years, and in the third six months. The indefinite and insidious way in which this disease progresses in the early stages is already well known, but that it may be ushered in by a lesion of the optic nerve in this way is a noteworthy fact.

Yet another fact bearing on the Ætiology of Optic Nerve Affections is brought out by Dr. J. Van de Hoeve, namely, the possibility of infection from the nasal sinuses. He publishes a case in which he was able to observe the development of an optic neuritis first on one side and then on the other, and by appropriate treatment was able to arrest the disease. The case is well worthy of record in point of diagnosis and also treatment. The patient, aged 24, first noticed that the vision of his left eye was defective, and consulted an oculist, who diagnosed retro-bulbar neuritis, and in the absence of any apparent cause supposed it to be specific. No good resulted from mercurial treatment, and Dr. Snellen was consulted. Visual acuity of left eye was then only 3/60, and there was a central scotoma. Inflammation of the sphenoidal sinus or posterior ethmoids was suspected, especially as the speech was somewhat nasal. Rhinoscopy revealed nothing definite. The right eye also became affected, and visual acuity was only 1/2. On both sides there was an atrophic sector of about 120° on the temporal side of the papilla, and on the left a central scotoma for white and all the colours except blue; on the right side there was considerable extension of the blind spot for most of the colours. The sphenoidal sinus and posterior ethmoidal cells of the left side were freely opened, and a little muco-pus escaped. Visual acuity of the left side improved rapidly, but on the right side the lesion progressed and a central scotoma developed. The same treatment was then repeated on the right side, and this was followed by still more marked improvement in the right vision than in the case of the left side. According to the author a central scotoma is one of the early symptoms of optic neuritis by infection from the nasal sinuses, and is preceded by an enlargement of the blind spot for colours as was actually observed in this case on the right side.

Dr. R. C. Rosenberger publishes in the *New York Medical Journal* some interesting research work on the Presence of the Tubercle Bacilli in the blood, by which he claims to have found the bacilli present in the blood in over 300 cases of tubercular disease of all kinds, including not only pulmonary tuberculosis, but also tubercular disease of the glands, bones and peritoneum, and in cases of tuberculous meningitis. It is recognised that in miliary tuberculosis the bacilli have found their way into the blood-stream, but according to this author all forms of the disease are of the nature of a "bacillæmia," and he goes so far as to suggest that the bacilli are probably present in the blood in a large number of apparently healthy individuals, that they remain there indefinitely in an attenuated condition, and become virulent at any time when the general resisting powers of the organism are diminished or any particular tissue or organ becomes injured, or otherwise affected. In this way he would explain the frequent occurrence of tubercular disease following all kinds of traumatism. He thinks also the bacilli may be transmitted from the maternal organism to the infant by means of the placenta. The details of his technique are as follows: 5 c.c. of blood is drawn from one of the veins of the arm under aseptic precautions, and is mixed with an equal amount of 2-per-cent. solution of potassium citrate in normal saline. The mixture is placed in a refrigerator for 24 hours, when a sediment is formed. This is spread on slides and dried with a moderate heat and then placed in sterilised distilled water, and fixed in the usual way over a Bunsen flame and stained.

Intravenous Chloroformisation is among the latest novelties in methods of anæsthesia. Arguing that the lungs are only intermediary agents and that the chloroform must be carried by the blood to the nerve centres in order to exercise its effect, Dr. Burkhardt of Wurtzbourg suggests that by introducing it directly into the circulation by intravenous injection, it might be possible to avoid accidents contingent to the pulmonary inhalation, reduce the dose, and adjust it more accurately. He has, therefore, carried out a number of experiments on cats, rabbits, and dogs, and has since also employed the method in four operations on the human subject with satisfactory results. An account of his work appears in the *Münchner Medizinische Wochenschrift*. He introduces the chloroform in normal saline solution, 100 c.c., containing 0.63 c.c. of chloroform. In animals he injected it into the jugular vein, and regulated the flow of the solution according to the corneal reflex. In this way he was able to keep the animals anæsthetised for a considerable period with a minimum amount of chloroform. He found that the concentration of chloroform in the blood sufficient to obtain narcosis amounted to 0.0415 per cent., which corresponds closely to the figures of Pohl for chloroform inhalation, namely, 0.015 to 0.043. The urine of some of the animals after the experiments showed the presence of albumen, and also cylindrical cells and hæmoglobin. The four patients were aged 79, 40, 14, and 13, and the operations were removal of an epithelioma of the hand, excision of tubercular glands of the neck, a plastic operation of the axilla, and a curetting of a tuberculous trochanter. The solution was injected into the median vein, complete anæsthesia was easily maintained, and there was only slight vomiting afterwards. In two cases there was albuminuria immediately afterwards, and in one case hæmoglobinuria, but these quickly disappeared. It is too early at present to say what advantages, if any, this method may offer over the more usual forms of anæsthesia.

---

It appears that both in America and in Saxony there have been cases of poisoning from the use of certain Hair Dyes, amongst which "Mrs. Potter's Walnut-Juice Hair Stain" has been mentioned in America. The toxic principle appears to be para-phenylene-diamine. According to a report issued by the North Dakota Agricultural Experimental Station, the proprietary article in question consists of two bottles: No. 1 contained 1.86 per cent. of absolute hydrogen peroxide; No. 2 was found to contain a substance conforming to the tests for para-phenylene-diamine in a solution containing 54.45 per cent. of absolute alcohol by volume. In one of the cases the symptoms consisted at first of an eruption similar to that of dermatitis venenata over the face, neck, and shoulders, running its course in about ten days. Then ensued a sub-acute attack of eczema involving the scalp, face, neck, trunk, and arms, persisting for six weeks and resisting treatment in a very stubborn way. In addition to the dermatitis there may be an extreme degree of itching, headache, and even swelling of the limbs and bloating of the face.

---

The treatment of Trigeminal Neuralgia by Schlösser's method apparently produces many good results. The essence of the technique is to inject a solution of alcohol into the affected branch of the nerve by means of a hypodermic needle and syringe. There is apt to be a little local pain for the time being; this is followed by anæsthesia which may last for a considerable time. If the pain should return owing to the regeneration of the nerve fibres, the procedure may be repeated. One formula for the fluid to be injected is as follows: Cocaine hydrochloride, 2 grains; chloroform, 10 mms.; absolute alcohol, 3 drachms; distilled water, ½ oz. Of this solution, 2 cubic centimetres are injected at a time. If a single injection relieves pain, well and good; if not, then the injection may be repeated after a couple of days; and if not by then, it may be given yet a third time at a corresponding interval.

---

A recent monograph by Lévy-Franckel discusses Chronic Aortitis and Aortic Atheroma in Children, with particular reference to those forms resulting from congenital syphilis. Clinical and pathological analysis of some 30 cases of aortic disease in children suggests that all the degrees of disease which occur in the adult can be recognised in the child, and that, moreover, it is easier to study the early stages of atheroma in the latter. The lesions appear to start in the middle coat of the vessel and to extend thence to the inner. Among the diseases which cause this degenerative process congenital syphilis easily stands first, though articular rheumatism, chorea, tuberculosis, scarlet fever, chronic nephritis, and myxœdema are responsible for a number of cases. Clinically he distinguishes four forms of aortic atheroma—(1) a variety in which the disease is latent; (2) pure aortic atheroma; (3) a form occurring with generalised arterio-sclerosis in other vessels; and (4) that associated with valvular lesions. In all cases diagnosis rests on the increased aortic dulness, the existence of a purring systolic thrill, and especially the presence of a rough systolic souffle heard at the base. Prognosis is bad, for the child is always exposed to the development of aortic aneurysm and to the danger of rupture of the aorta. These facts emphasise the necessity of applying vigorous antisyphilitic treatment from birth to all congenitally syphilitic children.

---

It is a matter of common knowledge that a certain proportion of new-born infants suffer from Noisy Respiration, most marked during the inspiratory act, and such cases are often classified as congenital laryngeal stridor. An article in the *Journal de Médécine Interne* from the pen of Terrien, discusses this affection at some length, and the author holds that it is not proper to regard the affection as arising from any single cause. Stridor, according to him, is a symptom which results from a number of pathological states, and he distinguishes the following types of stridorous respiration: (1) Congenital vestibular wheezing due to laryngeal deformity, in which the epiglottis is rolled in so that its lateral borders approach each other and the aryteno-epiglottidean folds are stuck together at their anterior thirds to form a kind of supplementary glottis, the vibrations of which give rise to the wheezing. (2) Stridor by spasm of the glottis. Too much weight must not, however, be given to the laryngeal examination, for at the moment of examination the child may contract its glottis and so produce a spasm which is, however, purely transitory. (3) Snoring due to adenoids, in which the bulky vegetations produce a laryngeal wheezing of a particular timbre which is exaggerated during sleep, and will often disappear, when the nasal fossæ are pinched together; this type of stridor is particularly intense, and the noise resembles grunting. (4) Tracheo-bronchial wheezing, rare in the infant, and distinguished by its tone, its persistence during expiration, and by the spasmodic cough which often accompanies it. (5) Stridor due to hypertrophy of the thymus. Here the wheezing is perceptible both during inspiration and expiration, and varies in intensity according to the position in which the head is held, being increased by extension and diminished by flexion. Radioscopic examination and careful percussion will moreover show that the thymus is enlarged.

---

In a recently published monograph, Dr. Murit, of Paris, points out that in the case of a pregnant woman attacked by a grave Pneumonia between the seventh and ninth month of gestation there is a likelihood of the disease spreading to the fœtus whether this latter be born prematurely or at term. The pneumococcus passes directly into the fœtal circulation through the placenta, and causes either generalised pneumococcal infections, such as septicæmia and pyæmia with multiple foci, or a localised pulmonary inflammation—*i.e.* a congenital pneumonia. In a few rare cases the child is born dead, but it usually lives a few hours or even a few days. The child breathes with difficulty, but auscultation reveals nothing abnormal. Signs of a grave infection are, however, present, such as prostration and a subicteric tinge of the integuments. In a short time the child becomes cold and shivers, the symptoms increase in severity, and death ensues with convulsions. Congenital pneumonia is not a common disease, since its occurrence necessitates the presence of a pneumococcal septicæmia in the mother, and the pneumococcus does not pass often into the circulation. Despite this relative rarity, the possibility of such an infection occurring must not be lost sight of in dealing with grave pneumonia in a pregnant woman, for the maternal prognosis is naturally rendered more serious by such an occurrence.

---

In the current issue of the *Journal of Tropical Medicine and Hygiene* appears a short paper by Dr. Nutford Atkinson, of Hong Kong, entitled "A Possible Natural Enemy to the Mosquito." The author describes a species of flower-fly, named *Lispa sinensis*, which, according to his observations, especially occupies itself in preying voraciously upon the larvæ of the mosquito tribe. The article is accompanied by illustrations of the fly's appearance and a short expert description by Mr. G. G. Austen, of the British Museum. Dr. Atkinson records certain observations made last summer in Hong Kong with reference to the devouring of larvæ by flies in the nullah and streams and stagnant waters of that colony. The flies which seemed then to be most active in the work of mosquito destruction were shortly afterwards recognised as being members of the *Dolichopodides*; but their full recognition has been deferred until the present summer. The flies brought home to England by Dr. Atkinson were all of the same species, and had been seen by him to possess a marked predilection for mosquito larvæ, which they devoured with extraordinary voracity and persistence. The author's preliminary report will, no doubt, be supplemented after his return to Hong Kong by further evidence in fuller detail as to the nature of the larvæ preyed upon by these aquatic flower-flies.

---

The cautious application of strong Pyrogallol vaseline ointment has been successful in a number of cases of Lupus Vulgaris. The treatment is commenced with a 10 per cent. ointment applied for several days. When vesicles form the strength of the ointment is reduced to 2 per cent. until grey granulations no longer appear. Then weaker applications are used, until a strength of only 0.1 per cent. is reached. If no improvement is evident with the 2 per cent. ointments a fresh application of 10 per cent. must be made for a day or two. The strong ointment should never be applied to the external ear, and for the nose one of 4 per cent. will generally be safer. The pain caused is not intolerable; morphine need only be employed with the 10 per cent. ointment, or when passing from a weaker to a stronger application. The ointment should be spread on the dressing with a wooden or vulcanite spatula. If a steel spatula is used the skin acquires an indelible grey or black stain. When dressing the lesions, the fresh dressing should be spread ready for use and applied immediately the old one is removed, since exposure of the treated surface to the air causes intense pain. Thirteen cases have been cured by this treatment.

---

Some recent work carried out by Déjean in France upon the relative strength of Colchicum preparations shows how much better in some respects it is to use the fresh than the dried corms. The fresh flowers are also very much preferred to the dried flowers whenever they are obtainable. The strong mother tincture made with fresh flowers, 1:1, of the 1884 Codex, is relatively much stronger than the tincture made with dried flowers—the therapeutic equivalent being 1: 12.8. Again the strong mother tincture of fresh flowers contains 0.768 parts of colchicine per 1,000, whereas that of the fresh corms contains 0.690 parts per 1,000. The relative pharmacological strengths of dried and fresh corms are as 1:19. This is evidently due to the fact that the alkaloids are deteriorated by the drying of the corm, so that in many galenical preparations it is clearly advisable to use the fresh drug.

---

It is not generally known how valuable a preventive against the bites of mosquitoes, fleas, gnats, midges, and so forth, Oil of Sassafras is. The fact has recently been recorded again by Mr. A. T. Girdler. If in a susceptible person the oil is applied at once to the place that has been bitten it almost invariably prevents the poisoning altogether. If applied to the inflamed spot a day or two after the bite it at once stops the irritation. To those who live in the country, and whose life is made a burden by undue susceptibility to insect bites, and to those who have not yet returned from holiday-making in regions infested by biting insects, Oil of Sassafras should be a great boon, and it is harmless as an external application.

